# Recognition of *Salmonella* by Dectin-1 induces presentation of peptide antigen to type B T cells

**DOI:** 10.1002/eji.201344065

**Published:** 2014-02-16

**Authors:** Nicola Jackson, Evan Compton, John Trowsdale, Adrian P Kelly

**Affiliations:** Division of Immunology, Department of Pathology, University of CambridgeCambridge, UK

**Keywords:** Antigen presentation, Autoimmunity, Bacterial infection

## Abstract

Type B T cells recognize peptide–MHC class II (pMHCII) isoforms that are structurally distinct from those recognized by conventional type A T cells. These alternative type B conformers result from peptide loading in the absence of HLA-DM. Type A conformers are more stable than type B pMHCII conformers but bind the same peptide in the same register. Here, we show that interaction of *Salmonella* Typhimurium with bone marrow derived dendritic cells (BMDCs) isolated from C3H/HeNCr1 mice results in enhanced presentation of peptide Ag to type B T cells. The effect could be mimicked by purified PAMPs, the most potent of which were curdlan and zymosan, β-(1,3)-glucan-containing polymers that are recognized by Dectin-1. Blocking of Dectin-1 with Ab and laminarin inhibited the induction of the type B T-cell response by BMDCs, confirming its role as a PRR for *S*. Typhimurium. Splenic DCs (sDCs) expressed Dectin-1 but were refractive to the induction of type B responses by *S*. Typhimurium and curdlan. Type B T cells have been shown to escape thymic tolerance and to transfer pathology in an autoimmune disease model. The induction of type B responses by gram-negative bacteria provides a mechanism by which autoreactive T cells may be produced during infection.

## Introduction

To protect the host from pathogens, MHC classc II (MHCII) molecules have evolved to present peptide Ag, derived from the invading organism, to CD4-restricted T cells. The MHCII processing pathway commences in the ER, where alpha and beta chains are folded and stabilized before transport into the endocytic pathway to complete assembly (reviewed in 1). Invariant chain plays an essential role in this process by providing a surrogate peptide, CLIP, to facilitate folding while in the ER. It also provides the targeting information required to direct the partially assembled MHCII molecule into endocytic compartments 2. In MHCII compartments, Ii is cleaved and CLIP exchanged for antigenic peptide under the direction of the MHCII-related molecule HLA-DM 3. Therefore, MHCII Ag processing requires the coordinated interaction of a range of molecules including MHCII, Ii chain, HLA-DM, and HLA-DO. These must be expressed in a regulated fashion and targeted to the correct intracellular compartments for efficient peptide loading.

For a given peptide–MHCII complex, different conformational isomers may be formed 4–7. These are structurally distinct and can be recognized by different T-cell subsets. Unanue et al. have characterized two groups of T cells, type A and type B, which recognize a dominant epitope derived from hen egg lysozyme (HEL) in the context of mouse I-A^k^ 4,5,8. The conformer recognized by type A T cells is generated from native Ag or peptide in late endocytic compartments under the direction of HLA-DM. In comparison, type B T cells recognize a different conformer generated from peptide Ag that is formed in the absence of HLA-DM, either at the cell surface or in early endocytic compartments 9. A similar phenotype is seen in human cells where both Ab and T cells can distinguish between peptide loaded in the presence or absence of HLA-DM 10.

Type B T cells form a significant proportion of the peripheral population and may be self-reactive. They can escape negative selection and participate in the development of autoimmune disease such as diabetes 11. T cells with a signature similar to the type B phenotype have also been identified in patients with myasthenia gravis 12 and multiple sclerosis 13, suggesting that the generation of these unconventional MHCII conformers may be linked to the pathology of multiple autoimmune diseases 14.

We have previously shown that infection of professional APCs with *Salmonella* Typhimurium results in enhanced ubiquitination of MHCII and reduced surface expression 15,16. We demonstrated that exposure to live or heat-killed *S*. Typhimurium (HKST) led to reduced presentation of peptide and native Ag to type A T cells. Presentation of peptide to type B T cells was dramatically increased, but presentation of native Ag was negligible 17. Total levels of HLA-DR and HLA-DM were unaffected by exposure to *S*. Typhimurium, implying that exposure altered the trafficking and distribution of pMHCII (peptide–MHCII) 16. In this investigation, we show that exposure to *S*. Typhimurium results in increased presentation of peptide Ag to type B T cells and identify PAMPs and DC subsets that participate in this process.

## Results

### Stimulation of BMDCs with microbial PAMPs induces the formation of type B MHCII conformers

**Figure 1:**
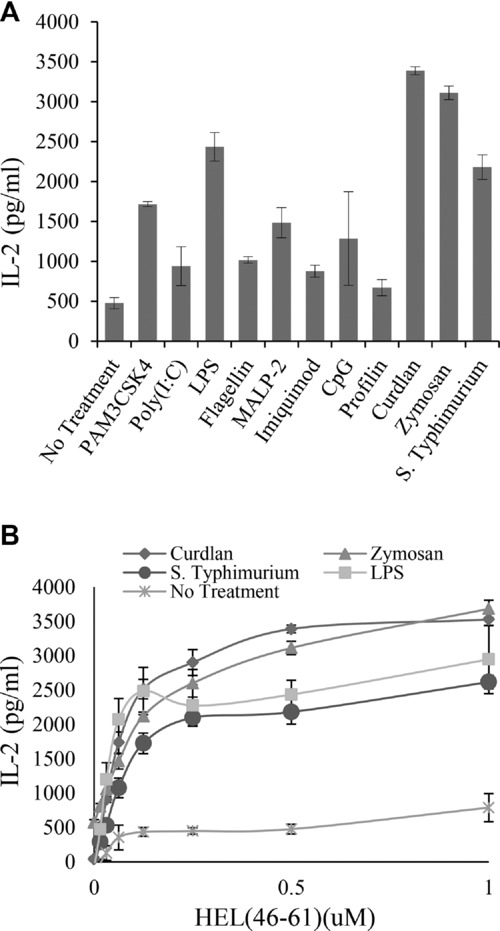
Exposure of BMDCs to *S*. Typhimurium and purified PAMPs enhances presentation to type B T cells. (A) BMDCs were exposed to HKST at a ratio of 50:1 (bacteria:BMDC) or one of a range of PAMPs (Pam_3_CSK_4_, 100 ng/mL; poly(I:C), 100 ng/mL; LPS, 1 μg/mL; flagellin,100 ng/ml; MALP-2, 100 ng/mL; Imiquimod, 5 μg/mL; CpG, 5 μg/mL; profilin, 0.5 μg/mL; curdlan, 100 μg/mL; and zymosan, 100 μg/mL) and presentation of HEL_46–61_ peptide to type B hybridoma (11A10) cells was measured by IL-2 production, as assessed by ELISA. Data shown are the mean ± SD of three samples from one of two independent experiments with similar results. (B) BMDCs were exposed to either curdlan, zymosan, LPS, or *S*. Typhimurium at the concentrations indicated in (A) overnight and presentation of HEL_46–61_ to type B hybridoma (11A10) cells was measured by IL-2 production, as measured by ELISA. Data shown are the mean ± SD of three samples from one of seven independent experiments with similar results.

We have previously shown that exposure of bone marrow derived dendritic cells (BMDCs) to *S*. Typhimurium results in an increase in presentation of exogenous peptide to type B T cells 17. The effect required direct contact between the bacteria and APC 17. To determine if the phenomenon was induced through recognition by PRRs, we exposed murine BMDCs to a panel of PAMPs and examined presentation to the type B T-cell hybridoma, 11A10. As shown in Figure1A, exposure to a diverse range of PAMPs increased peptide-dependent activation of type B T cells, but to varying degrees depending upon the PAMP. We monitored MHCII and CD80 levels on BMDCs after PAMP exposure. Similar levels of MHCII and CD80 expression were seen after stimulation with PAMPs that differed greatly in their ability to alter presentation of peptide to type B T cells (Supporting Information Fig. 1). Additionally, differences in presentation are unlikely to be due to CD80/86 expression, as presentation to 11A10 (type B) and 3A9 (type A) hybridomas is independent of costimulation 18. The most potent activators of presentation were zymosan and curdlan, two related PAMPs that are recognized by the C-type lectin Dectin-1. The dose–response titration for the most potent inducers of the type B response is shown in Figure1B. Dectin-1 binds to β-(1,3)-glucans expressed by fungi and some bacteria. Increased presentation was also seen in the presence of LPS, Pam_3_CSK_4_ (a synthetic triacylated lipopeptide), and macrophage-activating lipopeptide 2 (MALP-2). Therefore, recognition through Dectin-1 and a range of TLRs resulted in increased presentation of peptide Ag to type B T cells. The generation of type B MHCII conformers from HEL protein is negligible in BMDCs and is not induced by *S*. Typhimurium 17 or purified PAMPs (data not shown).

**Figure 2:**
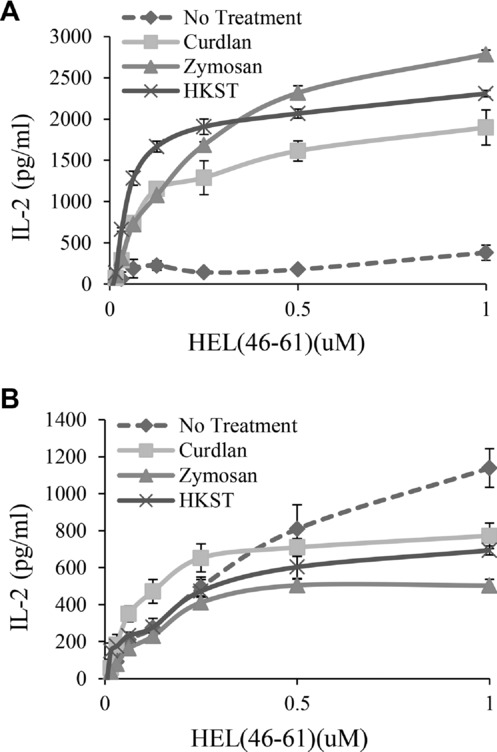
Presentation of HEL_46–61_ peptide to type A (3A9) and type B (11A10) T-cell hybridomas. (A) BMDCs were exposed to *S*. Typhimurium, curdlan, or zymosan at the concentrations indicated in Figure1 for 18 h. Cells were washed and then type B T cells (11A10) and various concentrations of HEL_46–61_ peptide were added for 24 h. Presentation of peptide was measured by IL-2 production as assessed by ELISA. (B) BMDCs were stimulated as in (A), but HEL_46–61_ peptide was presented to type A T cells (3A9) and IL-2 production was assessed by ELISA. Data in A and B show mean ± SEM from three samples from one of three independent experiments with similar results.

As the β-(1,3)-glucan-containing PAMPs induced the most significant alteration in presentation to type B T cells, we focused our investigation on these. We first determined if the increased presentation of peptide was specific to type B T cells. BMDCs were exposed to HKST, curdlan, or zymosan and presentation of exogenous peptide to type A (3A9) and type B (11A10) T cells was compared (Fig.2). All treatments induced expression of the costimulatory molecules CD80/86 to similar levels (data not shown). As before, increased presentation to type B T cells was observed in the presence of all three stimuli (Fig.2A), while presentation of peptide to the type A T cells (3A9) showed some reduction at higher peptide concentrations but was not grossly affected (Fig.2B). Therefore, the increased presentation of peptide to type B T cells cannot simply be explained by upregulation of costimulatory molecules.

### Recognition of *Salmonella* by Dectin-1 induces the formation of type B MHCII conformers

**Figure 3:**
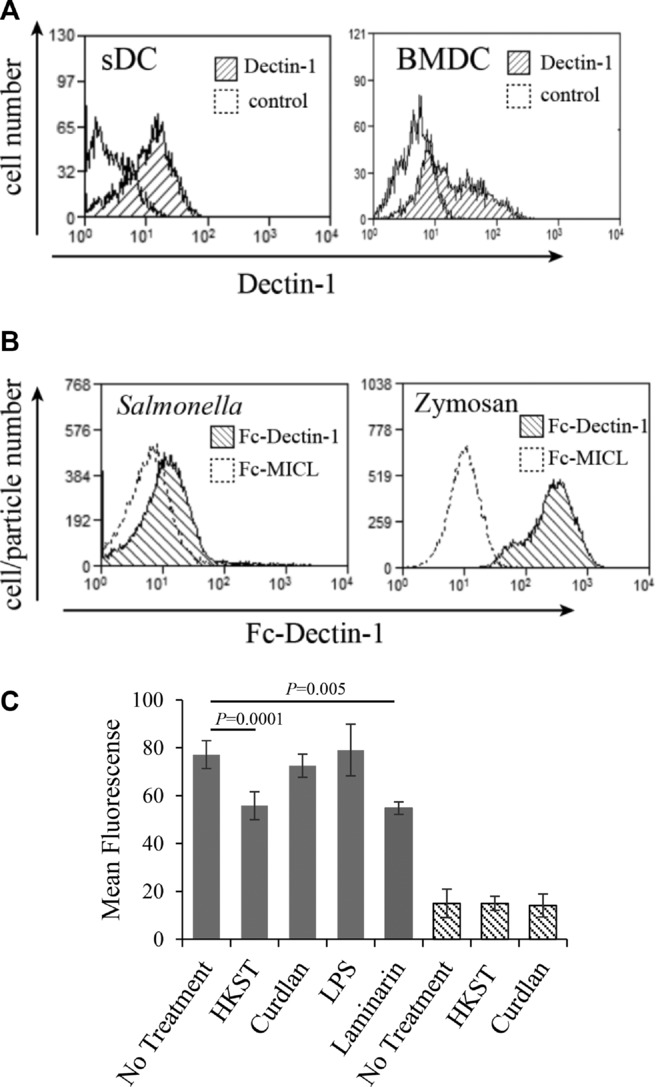
Dectin-1 expression on BMDCs is reduced upon interaction with HKST or laminarin. (A) Dectin-1 expression on CD11c^+^ BMDCs and sDCs was measured by flow cytometry using FITC-conjugated 2A11 anti-Dectin-1 Ab. Live cells were first gated on forward and side scatter and CD11c^+^ cells identified using CD11c-PE-Cy5. Histograms show Dectin-1 expression on CD11c^+^ cells. Plots are representative of six independent experiments with similar results. (B) The presence of β-(1,3)-glucan-containing ligands on *S*. Typhimurium was assessed by measuring FcDectin-1 binding to HKST. *S*. Typhimurium and zymosan were stained with the FcDectin-1 or control FcMICL fusion proteins at a concentration of 10 μg/mL. Zymosan particles and *S*. Typhimurium were gated on forward and side scatter and binding to FcDectin-1 or FcMICL detected with PE conjugated goat anti-human IgG by flow cytometry. Plots are representative of two independent experiments with similar results. (C) BMDCs and sDCs were exposed to HKST (50:1), curdlan (100 μg/mL), LPS (1 μg/ml), or laminarin (500 μg/mL) for 18 h and surface expression of Dectin-1 was measured by flow cytometry using the anti-Dectin-1 Ab 2A11. Data are shown as mean ± SD of samples pooled from six independent experiments. *p*-Values were calculated using a Student's paired *t*-test.

As polarization toward a type B T-cell response was induced by *S*. Typhimurium and ligands for Dectin-1, we examined whether the *S*. Typhimurium response was activated through Dectin-1. We first confirmed the presence of Dectin-1 on freshly prepared BMDCs by flow cytometry (Fig.3A). CD11c-positive cells presented as a bimodal population of cells that expressed low or high levels of Dectin-1. We then employed both direct and indirect assays to detect the *S*. Typhimurium/Dectin-1 interaction. We used a recombinant FcDectin-1 fusion protein to detect the presence of β-(1,3)-glucans on *S*. Typhimurium. We first confirmed that the reagent bound to zymosan, a yeast cell wall protein–carbohydrate preparation that contains repeating glucose units connected by β-(1,3)-glycosidic linkages. FcDectin-1 bound strongly to zymosan while a control Fc reagent did not (Fig.3B). FcDectin-1 also bound to HKST but at lower levels than to zymosan (Fig.3B).

We then followed the interaction indirectly using a receptor internalization assay. Contact with soluble ligand induces Dectin-1 receptor activation and internalization 19. We exposed BMDCs to *S*. Typhimurium and observed a reduction in the level of surface Dectin-1 (Fig.3C). As published, exposure to curdlan, which is too large to be phagocytosed, did not alter the level of surface Dectin-1, but exposure to the soluble ligand laminarin induced levels of receptor downregulation similar to *S*. Typhimurium (Fig.3C). Activation of DCs by LPS, which also induced enhanced presentation to type B T cells, did not influence Dectin-1 surface expression. From this we conclude that *S*. Typhimurium interacts directly with Dectin-1 present on BMDCs.

**Figure 4:**
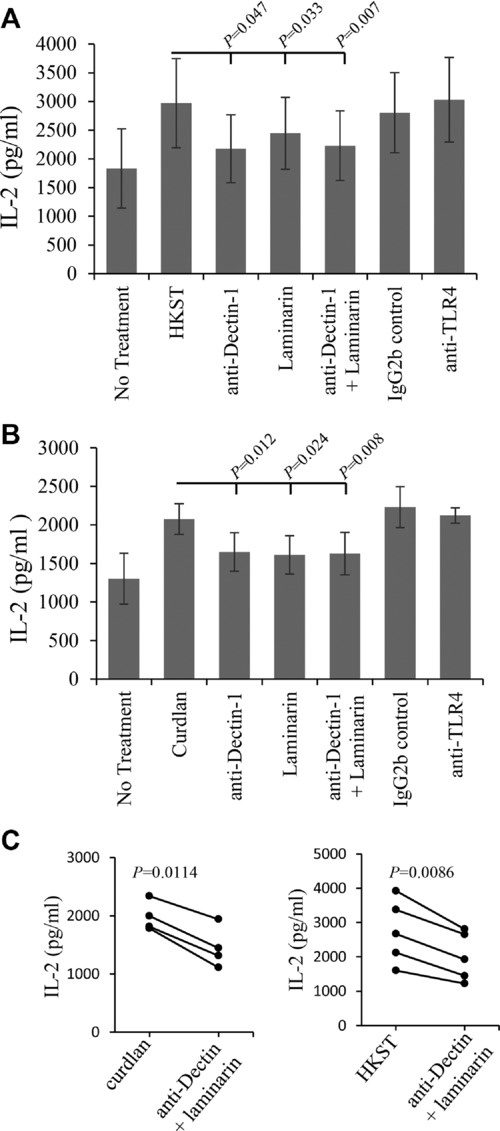
*Salmonella* and curdlan-induced peptide presentation to type B (11A10) T cells is blocked by anti-Dectin-1 Ab and laminarin. (A) BMDCs were either directly exposed to HKST or preincubated for 1 h with anti-Dectin-1 (2A11) Ab (25 μg/mL), laminarin (500 μg/mL), or anti-Dectin-1 Ab 2A11 (25 μg/mL) and laminarin (500 μg/mL) combined. After 24 h, presentation of HEL_46–61_ peptide to type B hybridoma (11A10) cells was measured by IL-2 production as described in Figure1. Data are shown as mean ± SEM of 12 samples pooled from four independent experiments. (B) BMDCs were treated as in (A), but exposed to curdlan (100 μg/mL) in place of HKST. Data are shown as mean ± SEM of nine samples pooled from four independent experiments. (C) Comparison of the efficiency of combined laminarin and anti-Dectin Ab blocking upon curdlan and HKST induced peptide presentation to type B T cells plotted as paired datasets. *p*-Values were calculated using Student's paired *t*-test.

To confirm the interaction, we also employed blocking with a Dectin-1-specific Ab reagent (2A11) and laminarin, a soluble β-(1,3)-glucan. As shown in Figure4B, the increase in presentation to type B T cells induced by curdlan was significantly blocked by the anti-Dectin-1 Ab 2A11 (63% inhibition), laminarin (55% inhibition), and anti-Dectin-1 Ab and laminarin in combination (66% inhibition). These reagents also inhibited the response induced by *S*. Typhimurium where anti-Dectin-1 Ab, laminarin, and combined treatment reduced the presentation to type B T cells by 55, 42, and 61%, respectively (Fig.4A). In all experiments, after stimulation with either curdlan or *S*. Typhimurium, blocking with anti-Dectin-1 Ab and laminarin alone or in combination resulted in reduced presentation to type B T cells. As the blocking with anti-Dectin-1 Ab alone is similar for stimulation with *S*. Typhimurium and curdlan (55 and 63%), this suggests that a major component of the stimulation by *S*. Typhimurium is via Dectin-1. To visualize the results of individual experiments, the combined blocking with 2A11 and laminarin are depicted graphically (Fig.4C). In all cases, blocking resulted in reduced presentation to type B T cells. Together, these studies show that Dectin-1 is expressed on BMDCs and that it can recognize β-(1,3)-glucan present on *S*. Typhimurium, curdlan, and zymosan. This recognition results in enhanced presentation of peptide Ag to type B T cells.

### *S.* Typhimurium induces the formation of type B MHCII conformers on BMDCs but not splenic DCs

Using splenic DCs (sDCs), Strong et al. investigated the influence of PRR activation on Ag presentation to type A and B T cells 18. They found no significant difference in the presentation of peptide Ag to either type A or type B T cells upon activation with a range of PAMPs including zymosan, LPS, CpG, and poly (I:C). We confirmed that sDCs express Dectin-1 (Fig.3A). Dectin-1 expression on BMDCs resolved cells into two populations, Dectin-1^hi^ and Dectin-1^lo^, with approximately 50% of cells in each category (Fig.3A). sDCs also expressed Dectin-1 but at levels intermediate of those seen in BMDCs. Stimulation with *S*. Typhimurium or curdlan did not alter Dectin-1 surface expression on sDCs (Fig.3C). As has also been previously reported 18, sDCs do not show the enhanced presentation of peptide to type B T cells (data not shown). We sorted populations of Dectin-1^hi^ and Dectin-1^lo^ expressing BMDCs and compared their ability to present to type B T cells. Both populations presented well to 11A10 cells (data not shown). Therefore, different DC subsets vary in their ability to present peptide to type B T cells and lack of presentation by sDCs is unlikely to be due to levels of Dectin-1 expression.

### Generation of type B MHCII conformers by different microbial pathogens

**Figure 5:**
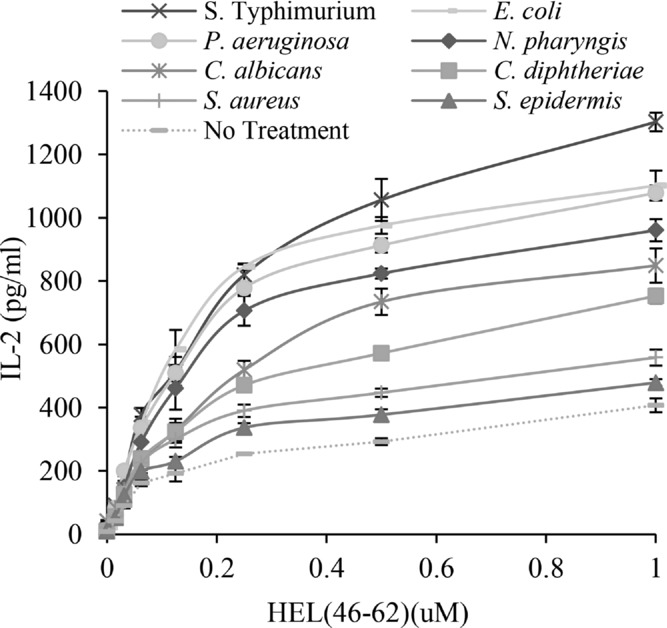
Gram-negative bacteria efficiently enhance presentation of HEL_46–61_ peptide to type B T (11A10) cells. BMDCs were exposed to the gram-negative bacteria *S*. Typhimurium, *E. coli, P. aeruginosa*, *or N. pharyngis*; gram-positive bacteria *S. aureus, S. epidermidis*, *or C. diphtheriae* or the yeast *C. albicans*. Presentation of HEL_46–61_ peptide to type B hybridoma (11A10) cells was then quantified as described in Figure1. Data are presented as mean ± SD of three samples from one of three independent experiments with similar results.

Finally, we investigated whether our observations were specific to *S*. Typhimurium or if exposure to other bacteria also resulted in altered presentation to type B T cells. BMDCs were exposed to yeast and a range of gram-positive and gram-negative bacteria, and presentation to type B T cells was examined. In order of potency, gram-negative bacteria induced the largest response, with *S*. Typhimurium being most effective followed by *Escherichia coli*, *Pseudomonas aeruginosa*, and *Neisseria pharyngis*. The yeast *Candida albicans* and *Corynebacterium diphtheriae* also enhanced presentation but to a lesser degree. Finally, the pyogenic gram-positive bacteria *Staphylococcus aureus* and *Staphylococcus epidermidis* had only a minor influence on presentation to type B T cells (Fig.5).

## Discussion

We show that exposure of BMDCs to *S*. Typhimurium induces the presentation of peptide Ag to type B T cells. This response is not simply the result of enhanced costimulatory capacity of the DCs, as there is no enhanced presentation to type A T cells. Additionally, the hybridomas used do not require costimulation 18. The effect could be mimicked by a number of purified PAMPs, the most potent of which were curdlan and zymosan. These β-(1,3)-glucan-containing ligands are recognized by Dectin-1 and are more commonly associated with fungal rather than bacterial recognition. We sought confirmation of the interaction using several complementary approaches. We demonstrated that (i) BMDCs expressed Dectin-1; (ii) *S*. Typhimurium expressed ligands that could be bound by a Dectin-1 Fc fusion protein; (iii) the response could be inhibited by laminarin, a soluble β-(1,3)-glucan blocking reagent, and by a Dectin-1 blocking Ab; (iv) *S*. Typhimurium induced internalization of surface expressed Dectin-1. Taken together, the data provide strong evidence that signaling through Dectin-1 can skew presentation of peptide toward a type B T-cell response. Altered peptide presentation was not limited to stimulation via curdlan and zymosan but was also induced by several other PAMPs including LPS, which is recognized by the TLR4/MD-2 activation complex; Pam_3_CSK_4_, a synthetic triacylated lipopeptide recognized by TLR1 and TLR2; and MALP-2, a TLR 2/6 agonist. Therefore the effect was induced by PAMPs that interact with both TLR and C-type lectin receptors, which function through different signaling pathways. In the case of exposure to *S*. Typhimurium, our blocking studies suggest that the response through Dectin-1 is dominant. Additional studies will be required to determine if the TLR PAMPs influence presentation to type B T cells when delivered at more physiological levels.

How can recognition of *S*. Typhimurium induce the generation of epitopes recognized by type B T cells? The HEL_46–61_ peptide binds I-A^k^ using an aspartate residue at position 52 in the P1 pocket 8. Both type A and type B T cells recognize this HEL_46–61_ peptide in a single register. Native HEL Ag requires degradation in late endocytic compartments and occurs in the presence of HLA-DM. This promotes the formation of minimum energy conformers recognized by type A T cells and limits the generation of less stable type B conformers 8. In contrast, peptide can load in early endocytic compartments independently of interaction with HLA-DM 9 leading to enhanced formation of the type B conformer. Therefore recognition of *S*. Typhimurium by DCs likely promotes the formation of pMHCII complexes in an HLA-DM-independent manner. DCs internalize Ag by multiple mechanisms including phagocytosis and macropinocytosis, processes that are enhanced by PRR signaling 20. Surface recognition of pathogens by TLR2, TLR4, and Dectin-1 accelerates rates of phagocytosis 20,21 and can influence soluble Ag uptake by macropinocytosis 22 and targeting to distinct MHCII-loading compartments 21. We propose that recognition of *S*. Typhimurium by PRRs directs changes in Ag/MHCII trafficking in BMDCs facilitating DM-independent peptide loading of MHCII.

We compared BMDCs with sDCs isolated from the same mice for their response to type B T cells. sDCs failed to show enhanced presentation to type B T cells when stimulated with *S*. Typhimurium, curdlan, or zymosan. They also failed to show downregulation of surface Dectin-1 when exposed to *S*. Typhimurium or curdlan. We looked for differences in presentation of sorted populations of Dectin-1^hi^ and Dectin-1^lo^ expressing BMDCs but found that they were equally efficient at presenting peptide to type B T cells. Therefore, the level of Dectin-1 expression cannot explain the lack of presentation by sDCs. Strong et al. have previously examined the influence of PAMPs, including LPS and zymosan, on presentation of native HEL and HEL_46–61_ by sDCs. They concluded that there was no significant increase in presentation of HEL_46–61_ peptide to type B T cells upon PAMP stimulation 18. Our data confirm this and we conclude that different DC populations vary in their ability to handle and load exogenous peptide in an HLA-DM-independent fashion. The different behavior of sDC and BMDC populations may relate to “priming” by exposure to GM-CSF. Primary macrophages that fail to respond to β-glucans through Dectin-1 can be reprogrammed to do so by exposure to GM-CSF 23. GM-CSF is also an important cytokine with respect to DC lineage development and is known to alter the Ag-presentation properties of cells.

In future studies, it will be important to investigate responses by additional DC and macrophage populations and the potential influence of cytokines including GM-CSF on priming of responses in these cells. We considered how relevant our observations might be to a broad range of infections by examining whether additional microbial species also modified presentation to type B T cells. Gram-negative species, *S*. Typhimurium in particular, were most efficient at inducing the type B conformer. Yeast and some gram-positive bacteria were also able to induce the response, albeit at lower efficiency. Therefore, microbial interaction with BMDCs in general generated type B conformers. Type B T cells constitute a significant proportion of the responder T-cell population (30–50%) and these cells have been implicated in a number of autoimmune diseases (reviewed in 14). Activation of BMDCs in the presence of these PAMPs may in part explain the prevalence of the type B conformer in the circulation. This study identifies BMDCs as a population of APCs capable of inducing activation of type B T cells.

In the context of *S*. Typhimurium, ligands for Dectin-1 appear particularly important for the generation of type B pMHCII conformers on BMDCs. Priming of TLR responses through associated activation of Dectin-1 by β-glucans has been established and is proposed to be a mechanism to enhance TLR-mediated signaling 24. Signals through Dectin-1, TLRs and possibly additional PRRs, likely combine to enhance HLA-DM-independent MHCII peptide loading thereby increasing expression of type B pMHCII conformers. These alternative MHCII isomers could be protective or harmful. Their existence implies a beneficial function and as such they have been suggested to represent an evolutionarily conserved mechanism for augmenting T-cell responses to pathogen possibly by diversifying the peptide repertoire 8. The generation of type B responses in DCs would presumably be mimicked during B-cell activation by HLA-DO expression suggesting a role for type B MHCII conformers in regulating B-cell responses to pathogen. Their generation could occur in an inflammatory setting through acquisition of peptide or partially digested protein produced through the action of phagocytic cells and the loading of this material onto MHCII in an HLA-DM-independent fashion 8. During infection, this could expand the repertoire of peptides presented by MHCII. However, peptide could also derive from self-Ag and participate in the induction of autoreactivity 11. Enhanced endocytosis of exogenous peptide would also benefit cross-presentation. MHC class I molecules traffic from the cell surface into early and late endosomal compartments and can recycle back to the cell surface. TLR engagement by DCs promotes cross-presentation to MHC class I restricted T cells 25 and it will be of interest to determine if recognition through Dectin-1 also facilitates this process.

## Materials and methods

### Antibodies

Antibodies used were the following: FITC-conjugated anti-Dectin-1 [2A11], GeneTex; Rat IgG2b K Isotype Control, eBioscience; CD11c PE-Cy5 (N418), eBioscience; anti-mouse Dectin-1 Ab 2A11, gift from Professor Philip Taylor (University of Cardiff, UK); PE anti-mouse CD80 (B7–1, clone: 16–10A1) eBioscience; PE anti-Mouse CD11b (Clone: M1/70), eBioscience; PE goat anti-human IgG Fc, Jackson Laboratories. FcDectin-1 and control reagent FcMICL were gifts from Professor Philip Taylor. For staining *S*. Typhimurium FcDectin-1 fusion protein was used at a concentration of 10 μg/mL.

### Bacteria and PAMPs

*S*. Typhimurium 12023 was from the ATCC. *E. coli, P. aeruginosa, N. pharyngis*, *S. aureus, S. epidermidis, C. diphtheriae*, and *C. albicans* were from the Department of Pathology, University of Cambridge. Heat-killed bacteria were freshly produced by heating cells at 70°C for 45 min and used at a ratio of 50:1 (bacteria:BMDCs). PAM_3_CSK_4_, Poly(I:C), LPS, flagellin, MALP-2, Imiquimod, CpG, and profilin were from Apotech; curdlan was from Alpha Laboratories and laminarin and zymosan were from Sigma.

### Flow cytometry

Cells were harvested using a cell scraper and incubated with appropriate antibodies in FACS buffer (PBS, 5% FCS) at 4°C. BMDCs were incubated with Mouse Fc Block (BD Biosciences) for 10 min at 4°C prior to addition of antibodies. After washing, cells were analyzed using a FACScan Flow Cytometer and Summit software (BD Biosciences). Student's *t*-test was performed using Microsoft Excel software. For purification of populations of Dectin-1^hi^ and Dectin-1^lo^ expressing BMDCs, cells were stained for Dectin-1 with Mab 2A11 and sorted using a MoFlo flow cytometer (Cytomation).

### BMDC preparation and Ag presentation

Mice (C3H/HeNCr1) were from the Charles River and were maintained according to institutional guidelines at the University of Cambridge. BMDCs were prepared as previously reported 17. Briefly femurs and tibias from C3H/HeNCr1 mice were defleshed and BM flushed into IMDM. Cells were dispersed by passage through a 70 um cell strainer, centrifuged, and seeded into 9 cm Petri dishes at 1 × 10^6^ cells/mL in IMDM 10% FCS, 2μM ultraglutamine, 10 ng/mL IL-4, and 20 ng/mL GM-CSF with penicillin and streptomycin. After 30 min, the nonadherent cells were recovered and reseeded into 6-well plates and matured for 7 days with media changes on days 3 and 5. Day 7 BMDCs were recovered by gentle scraping on ice. These differentiated cells were routinely 50–60% CD11c/CD11b^+^, CD80^hi^, CD86^lo^, and MHCII^lo^.

### Ag presentation

Ag presentation assays were performed in 96-well round-bottomed plates 17. Type A T-cell hybridoma 3A9 and type B T-cell hybridoma 11A10 were from Professor Emil Unanue (Washington, USA). Briefly, BMDCs were seeded at 3 × 10^4^ cells/well. Cells were exposed to HEL Ag or peptide (HEL_46–61_), with or without PAMP stimulants/*S*. Typhimurium for 18 h in 100 μl volume. The following concentrations of stimulants were used; HKST (50:1 ratio of bacteria:cells), 100 ng/mL PAM3CSK4, 100 ng/mL Poly(I:C), 1 μg/mL LPS, 100 ng/mL flagellin, 100 ng/mL MALP-2, 5 μg /mL imiquimod, 5 μg /mL CpG, 0.5 μg /mL profilin, 100 μg /mL curdlan, and 100 μg /mL zymosan. DCs were washed three times and 5 × 10^4^ washed T hybridoma cells (type-B 11A10 or type A 3A9) were added per well. Culture supernatants were harvested after 24 h and frozen at −80°C. Released IL-2 was quantified by ELISA using a mouse IL-2 Ready-SET-Go kit (eBioscience).

## Abbreviations

BMDC: BM-derived DC
HEL: hen egg lysozyme
HKST: heat-killed *S*. Typhimurium
sDC: splenic DC
MALP-2: macrophage-activating lipopeptide 2
MHCII: MHC class II
pMHCII: peptide–MHC class II
